# Tissue-specific root ion profiling reveals essential roles of the CAX and ACA calcium transport systems in response to hypoxia in Arabidopsis

**DOI:** 10.1093/jxb/erw034

**Published:** 2016-02-17

**Authors:** Feifei Wang, Zhong-Hua Chen, Xiaohui Liu, Timothy David Colmer, Meixue Zhou, Sergey Shabala

**Affiliations:** ^1^School of Land and Food, University of Tasmania, Hobart, Tasmania 7001, Australia; ^2^School of Science and Health, Western Sydney University, Penrith NSW2751, Australia; ^3^School of Chemical Engineering and Technology, Tianjin University, Tianjin 300072, China; ^4^School of Plant Biology and Institute of Agriculture, The University of Western Australia, Crawley, WA 6009, Australia

**Keywords:** Ca^2+^-ATPase, calcium, Ca^2+^/proton exchanger, confocal microscopy, epidermis, hypoxia, potassium, sodium, stele, waterlogging.

## Abstract

Waterlogging is a major abiotic stress that limits the growth of plants. The crucial role of Ca^2+^ as a second messenger in response to abiotic and biotic stimuli has been widely recognized in plants. However, the physiological and molecular mechanisms of Ca^2+^ distribution within specific cell types in different root zones under hypoxia is poorly understood. In this work, whole-plant physiological and tissue-specific Ca^2+^ changes were studied using several *ACA* (Ca^2+^-ATPase) and *CAX* (Ca^2+^/proton exchanger) knock-out Arabidopsis mutants subjected to waterlogging treatment. In the wild-type (WT) plants, several days of hypoxia decreased the expression of *ACA8*, *CAX4*, and *CAX11* by 33% and 50% compared with the control. The hypoxic treatment also resulted in an up to 11-fold tissue-dependent increase in Ca^2+^ accumulation in root tissues as revealed by confocal microscopy. The increase was much higher in stelar cells in the mature zone of Arabidopsis mutants with loss of function for *ACA8*, *ACA11*, *CAX4*, and *CAX11*. In addition, a significantly increased Ca^2+^ concentration was found in the cytosol of stelar cells in the mature zone after hypoxic treatment. Three weeks of waterlogging resulted in dramatic loss of shoot biomass in *cax11* plants (67% loss in shoot dry weight), while in the WT and other transport mutants this decline was only 14–22%. These results were also consistent with a decline in leaf chlorophyll fluorescence (*F*_v_/*F*_m_). It is suggested that CAX11 plays a key role in maintaining cytosolic Ca^2+^ homeostasis and/or signalling in root cells under hypoxic conditions.

## Highlight

Physiological and molecular mechanisms of cytosolic Ca^2+^ homeostasis and signalling in plant adaptive responses to hypoxia were investigated and the critical role of CAX11 exchangers in this process was revealed.

## Introduction

Waterlogging is a widespread abiotic stress to land plants, influencing nearly 10% of the global land area (Setter and Waters, 2003) and reducing up to 80% of crop yields (Shabala, 2011). There is a series of changes in physical, chemical, and biological properties in soil which ultimately inhibit growth of intolerant plants under waterlogging stress. The diffusivity of atmospheric oxygen (O_2_) into soil pores is reduced by ~10 000-fold under waterlogging, which exposes the plant roots to a hypoxic situation (Malik *et al.*, 2001; Armstrong and Drew, 2002). The O_2_ deficiency in the root zone depresses plant shoot and root growth, dry matter accumulation, and yield (Malik *et al.*, 2001; Colmer and Greenway, 2011; Shabala *et al.*, 2014). In addition, hypoxia limits the supply of ATP to plant H^+^-ATPase pumps and impairs ion transport processes, cell metabolism, and nutrient acquisition (Bailey-Serres and Voesenek, 2008; Elzenga and van Veen, 2010; Shabala *et al.*, 2014).

Cytosolic free Ca^2+^ ([Ca^2+^]_cyt_) has been widely recognized as the central regulatory signal for many processes in plant cells and as a key second messenger mediating plant adaptive responses to abiotic and biotic stimuli (Reddy *et al.*, 2011; Steinhorst and Kudla, 2014; Zhu *et al.*, 2015). These usually include multiple signal transduction pathways when plants are exposed to O_2_ deficiency, salinity, drought, osmotic, oxidative, heat, and cold stresses, pathogens, and bacterial and fungal signals (Sanders *et al.*, 1999; McAinsh and Pittman, 2009; Bose *et al.*, 2011). Hypoxic stress causes a rapid elevation of [Ca^2+^]_cyt_ in the cells of Arabidopsis, maize, rice, and wheat (Subbaiah *et al.*, 1994; Yemelyanov *et al.*, 2011; Lindberg *et al.*, 2012), and this elevation is fundamental for gene activation and acclimation responses at the cellular, tissue, as well as organismal levels (Subbaiah and Sachs, 2003; Shabala *et al.*, 2014).

Potassium is not only essential for many plant metabolic processes, such as osmoregulation, enzyme function, stomatal movements, control of membrane polarization, and ionic balancing (Dreyer and Uozumi, 2011; Wang *et al.*, 2013; Shabala and Pottosin, 2014), but also plays an important role in determining the fate of cells under stress conditions (Shabala, 2009; Demidchik *et al.*, 2010; Anschutz *et al.*, 2014; Demidchik *et al.*, 2014). K^+^ homeostasis is essential to mediate plant adaptive responses to a broad range of abiotic and biotic stresses including drought, salinity, and oxidative stress (Chen *et al.*, 2007; Cuin *et al.*, 2008; Shabala *et al.*, 2010; Bonales-Alatorre *et al.*, 2013a; Anschutz *et al.*, 2014; Demidchik, 2014). In rice seedlings, it was found that K^+^ effluxes in the coleoptile were down-regulated during prolonged anoxia (Colmer *et al.*, 2001). The waterlogging-sensitive barley cultivar Naso Nijo showed a larger decline of K^+^ uptake in the mature zone after hypoxic treatment than waterlogging-tolerant TX9425 and CM72 cultivars. Also, the overall magnitude of the reduction of K^+^ efflux in the elongation zone was significantly higher in Naso Nijo than in TX9425 and CM72 (Pang *et al.*, 2006; Zeng *et al.*, 2014). Therefore, a root’s ability to retain K^+^ under hypoxic conditions is strongly correlated with waterlogging tolerance in barley, and highlights the root tissue specificity of plant-adaptive responses to hypoxia (Zeng *et al.*, 2014). Salinity and waterlogging stress are abiotic stresses that often occur together in estuaries, river flood plains, agricultural land affected by secondary salinity (dryland and irrigated), or in areas irrigated with saline water (Smedema and Shiati, 2002; Qadir and Oster, 2004). When salinity occurs together with hypoxia, the transfer of Na^+^ and Cl^−^ from roots to shoots increases, whereas K^+^ transport decreases even further compared with salinity under normoxic conditions (Barrett-Lennard and Shabala, 2013). Very few studies have elaborated Na^+^ and K^+^ distribution in specific cell types during combined salinity and hypoxia stress.

As a ubiquitous denominator of cellular signalling networks, [Ca^2+^]_cyt_ signal is shaped by the balance of Ca^2+^ influx and efflux that is regulated by the membrane transport systems. The main Ca^2+^ sources entering into the cytoplasm are from the apoplast, vacuole, endoplasmic reticulum (ER), mitochondria, and chloroplast, through a large number of voltage-gated and ligand-gated Ca^2+^ channels at the plasma membrane, tonoplast, and endomembranes (Medvedev, 2005; Pottosin and Schnknecht, 2007; McAinsh and Pittman, 2009). Reducing [Ca^2+^]_cyt_ to the resting level requires activation of Ca^2+^ transporters such as high-affinity Ca^2+^-ATPases [autoinhibited Ca^2+^-ATPases (ACAs) and ER-type Ca^2+^-ATPases)] and the low-affinity Ca^2+^/H^+^ antiporter (CAXs) which are localized in both the plasma membrane and endomembranes (Sanders *et al.*, 2002; Hetherington and Brownlee, 2004; Manohar *et al.*, 2011; Huda *et al.*, 2013). The different subcellular locations of these Ca^2+^ circuits may contribute to the unique code of Ca^2+^ signals and provide a mechanism for creating a stimulus-specific Ca^2+^ signature (Boursiac *et al.*, 2010).

As [Ca^2+^]_cyt_ increases in response to environmental stress, the Ca^2+^ efflux system plays critical roles in restoring basal [Ca^2+^]_cyt_ levels and terminating stress-induced [Ca^2+^]_cyt_ signatures (Bose *et al.*, 2011; Shabala, 2011). Analysis of Arabidopsis mutants, in particular *aca4* and *aca8*, identified a variety of phenotypes including sensitivity to salt and cold stress, indicating these transporters play roles in plant stress responses (Geisler *et al.*, 2000; Schiott and Palmgren, 2005). In yeast and tobacco BY-2 cells, a Ca^2+^/H^+^ antiporter, CAX4, is found to be localized on the tonoplast, and the *CAX4* mRNA level increased after Mn^2+^, Na^+^, and Ni^+^ treatment (Cheng *et al.*, 2002). The loss-of-function mutant (*cax4-1*) and CAX4 RNA interference (*CAX4* RNAi) lines indicated altered root growth and development in response to Cd^2+^, Mn^2+^, and auxin (Mei *et al.*, 2009). These results demonstrated that CAX4 functions in root growth under heavy metal stress conditions. The function of CAX11 was also characterized in yeast, in which the fusion of CAX11 and green fluorescent protein (GFP) was found in the plasma membrane and nuclear periphery. Expressing CAX11 in a yeast mutant showed its role of mediating high-affinity K^+^ uptake and Na^+^ transport (Zhang *et al.*, 2011). However, the exact role of these transporters is still elusive.

An increase in Ca^2+^ concentration in the cytosol and nucleus is detected by many Ca^2+^-sensing proteins [e.g. calmodulin (CaM), calmodulin-like proteins (CMLs), Ca^2+^-dependent protein kinases (CDPKs), and calcineurin B-like (CBL)/Ca^2+^-independent protein kinases (CIPKs)] (Dodd *et al.*, 2010; Reddy *et al.*, 2011). These proteins are able to decode the information present within the different Ca^2+^ spikes or oscillations and process this information into alteration of the cell function. For example, ZmCAP1, encoding Ca^2+^-ATPase, from maize has been shown to bind to and be stimulated by CaM following heterologous expression in yeast (Subbaiah and Sachs, 2000). Following an elevation in [Ca^2+^]_cyt_, CaM will interact with an N-terminal autoinhibitory domain of the ACAs, leading to activation of Ca^2+^ transport activity (Baekgaard *et al.*, 2005). These CaM-binding domains appear to be ubiquitous on all ACAs, but are highly divergent with little consensus sequence (Chung *et al.*, 2000; Baekgaard *et al.*, 2006). Different signalling pathways are regulated by the transcription machinery and expression of downstream target genes. The downstream reactions can cause an acclimation, helping the plant to survive the specific stress (Lindberg *et al.*, 2012). Thus, it is of importance to carry out investigations and screening of the interaction of the Ca^2+^-sensing proteins with Ca^2+^-ATPase or Ca^2+^/H^+^ exchanger proteins under hypoxic stress.

Knowledge of hypoxia-induced transport processes is largely restricted to ion exchange at the root surface, which is easily accessible. However, little is known about the character of hypoxic signal transduction in specific cell types and tissues of roots (Pang *et al.*, 2006; Zeng *et al.*, 2014; Kotula *et al.*, 2015). We hypothesize that radial and longitudinal O_2_ profiles reported in plant roots under conditions of hypoxia have a major impact on the energy availability in various cell types, thus affecting their ionic homeostasis. We have also postulated that a lack of functional Ca^2+^ efflux systems may be detrimental to plant acclimation to hypoxic conditions. Both these hypotheses were confirmed in the current study. The objective of our research was to investigate anoxia-induced changes of Ca^2+^, K^+^, and Na^+^ in meristem, epidermal, and stelar cells of Arabidopsis in physiologically different root zones in wild-type (WT), and *aca* and *cax* mutants to reveal essential roles of the CAX and ACA calcium transport systems. Overall, our results suggest that Ca^2+^ efflux systems and especially CAX11 are essential to mediate plant Ca^2+^ homeostasis under hypoxic conditions.

## Materials and methods

### Plant materials and treatments

Seeds of *Arabidopsis thaliana* WT Columbia-0 (Col-0) and loss-of-function mutants (all in the Col-0 background) *aca8* (SALK_0578770), *aca11* (SALK_002747), *cax4* (SALK_119863), and *cax11* (SALK_013040) were obtained from the Arabidopsis Biological Resource Centre (http://www.arabidopsis.org/abrc/). Seeds were surface sterilized with 1ml of commercial bleach [1% (v/v) NaClO] for 10min, and then washed five times with sterilized distilled water. Seeds were kept at 4 °C for 2 d and sown in Petri dishes containing 1% (w/v) phytogel, half-strength Murashige and Skoog (MS) medium, and 0.5% (w/v) sucrose at pH 5.7. Petri dishes containing seeds were sealed with 3M micro-pore tape and then transferred into a growth chamber with a 16h/8h day/night, 100 µmol m^−2^ s^−1^ photon flux density, and 22 °C. The Petri dishes were oriented upright, allowing the roots to grow down along the surface without penetrating into the medium. All chemicals were from Sigma-Aldrich (Castle Hill, NSW, Australia) in analytical grade unless individually specified.

Hypoxic treatment was imposed on the 10-day-old Arabidopsis seedlings submerged with 0.2% (w/v) agar solution pre-bubbled with high purity N_2_ (Coregas, Sydney, NSW Australia). For measurement of gene expression levels, WT Arabidopsis seedlings were treated with hypoxia for 1, 24, and 72h; for measurement of K^+^ and Ca^2+^ distribution in roots, the WT and the four mutants were treated with hypoxia for 24h. Combined hypoxia and salinity treatment was also imposed on the 10-day-old Arabidopsis seedlings in deoxygenated agar solution with addition of 50mM NaCl for 24h, with measurement of Na^+^ distribution in roots. The whole seedlings were submerged in the bubbled solution and kept in the growth chamber with a 16h/8h day/light, 100 µmol m^−2^ s^−1^ photon flux density, and at 22 °C. Compared with hypoxia treatment or combined hypoxia and salinity treatment, the control condition was normoxic, non-saline, and without agar solution.

In the phenotyping experiments, *aca8*, *aca11*, *cax4*, c*ax11*, and WT plants were grown in 0.2 litre pots filled with peat moss, perlite, vermiculite, and coarse sand, with a ratio of 2:1:1:1 (v/v), and watered with half-strength Hoagland’s nutrient solution. Plants were grown in a growth chamber at a 12h/12h light/dark regime and at 21 °C for 3 weeks. Plants were then subjected to waterlogging treatment by immersing pots in water and maintaining the water level 0.5cm above the soil surface for another 3 weeks. The above-ground biomass of individual plants was determined as fresh weight, and dry weight was measured after drying in a Unitherm Drier (Birmingham, UK) for 2 d at 65 °C.

### Chlorophyll fluorescence

Chlorophyll fluorescence was measured on the 6-week-old fully expanded leaves which had been subjected to the 3 week treatment with an OS-30p chlorophyll fluorometer (OPTI-Sciences, Hudson, USA). Plants were adapted in a dark chamber for 30min before measuring. The maximum quantum efficiency of photosystem II (PSII; *F*_v_/*F*_m_) was recorded at a saturating actinic light (660nm) intensity of 1100 µmol m^−2^ s^−1^ (Zeng *et al.*, 2013). Measurements were taken in at least five replicates for each treatment.

### Quantitative real-time PCR (RT-PCR)

Total RNA was extracted from 10-day-old Arabidopsis roots with TRIZOL reagent (Life Technologies, Mulgrave, VIC, Australia) (Liu *et al.*, 2014; Chen *et al.*, 2016). First-strand cDNA synthesis was performed using the sensiFAST Kit (Bioline, Alexandria, NSW, Australia) with 1 µg of total RNA. RT-PCR was performed with SensiMix SYBR No-ROX Kit (Bioline, Alexandria, NSW, Australia) using a Rotor-Gene Q6000 (QIAGEN, Hilden, Germany). The specific primers are listed in Supplementary Table S1 at *JXB* online. The PCR program was two steps: one cycle of 95 °C, 10min; and 40 cycles of 95 °C, 15s; 60 °C, 15s; and 72 °C, 15s. The amplification of the target genes was monitored every cycle by SYBR-green fluorescence. Three technical and biological replicates were performed for each experiment and treatment. The transcripts of target genes were normalized to the control gene RNA polymerase II subunit (*RPB2*).

### Confocal laser scanning microscopy measurements

CoroNa Green acetoxymethyl (AM) ester (Invitrogen, Eugene, OR, USA), Calcium Green-5N, AM (Invitrogen), and Asante Potassium Green-2 (APG-2, TEFLabs, Austin, TX, USA) were employed to measure the Na^+^, Ca^2+^, and K^+^ concentration, respectively, in Arabidopsis root cells. The optimal CoroNa Green concentration was determined in our previous work (Bonales-Alatorre *et al.*, 2013b; Wu *et al.*, 2015). All five types of root cells showed optimal fluorescence intensity when exposed to 15 µM Calcium Green-5N (Supplementary Fig. S1) and 20 µM APG-2. The indicators were dissolved in DMSO (Sigma) to a stock concentration of 1mM. Then, 20 µM CoroNa Green, 20 µM Asante Potassium Green-2, and 15 µM Calcium Green-5N were added to measuring buffers (Na^+^, 10mM KCl, 5mM Ca^2+^-MES, pH 6.1; K^+^, 5mM NaCl, 5mM Ca^2+^-MES, pH 6.1; Ca^2+^, 10mM KCl, 5mM Na^+^-MES, pH 6.1), respectively. Arabidopsis seedlings were incubated in the dye-containing measuring buffers for 3h in the dark. The stained seedlings were washed in distilled water for 3min to remove residual dyes before measuring fluorescence intensity in meristem, elongation epidermal, elongation stelar, mature epidermal, and mature stelar root cells after 24h of hypoxic treatment. All dying steps in this work was done at room temperature.

**Fig. 3.**
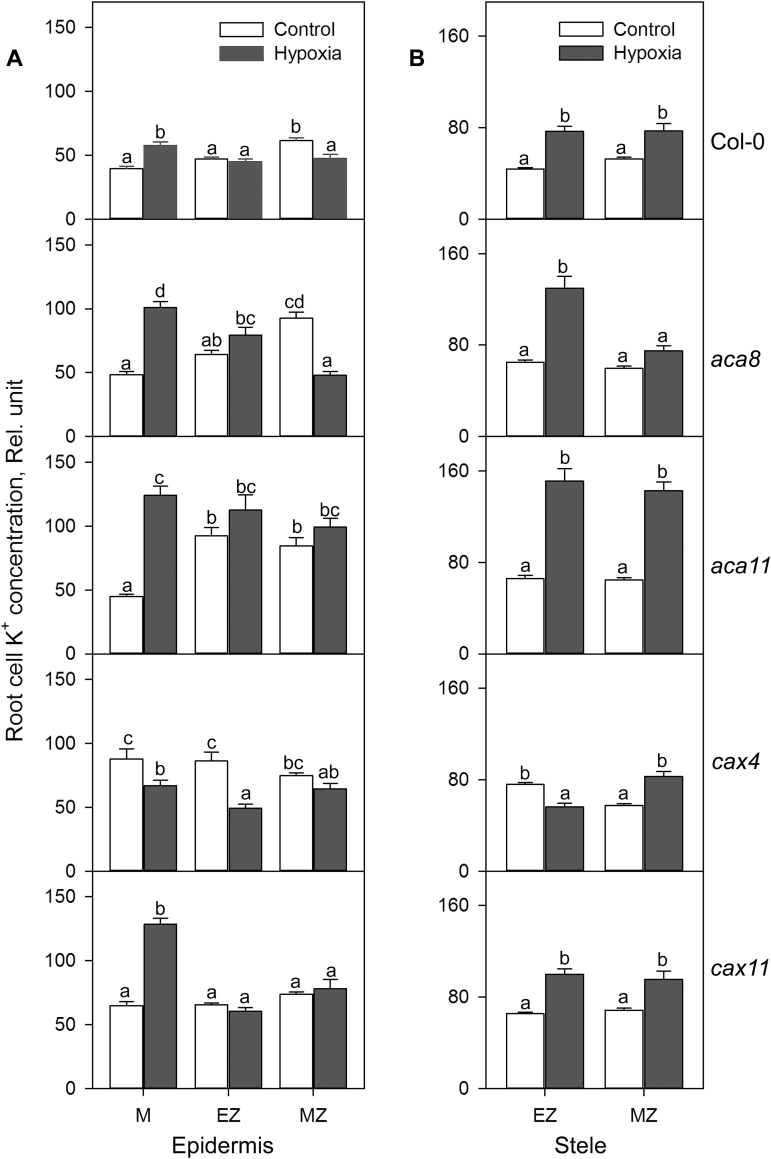
K^+^ concentrations in the meristem, elongation, and mature zone in root epidermis (A) and stele (B) of Col-0, *aca8*, *aca11*, *cax4*, and *cax11* under normoxic or hypoxic treatments for 24h. The relative K^+^ concentration was calculated by the fluorescence integrated density using Image J software. Data are the mean ±SE (*n*=30–40 cells from one individual plant with at least nine replicate plants). Different lower case letters indicate significant differences at *P*<0.05.

A confocal laser scanning microscope fitted with a TCS SPII confocal head (SP5, Leica Microsystems, Heidelberg, Germany) was used to measure fluorescent signals from roots. We used the 488nm excitation line of an argon multiline laser and the tripledichroic TD 488/543/633nm beam splitter. CoroNa Green fluorescence emission was detected in the photomultiplier at 505–525nm. Calcium Green-5N fluorescence emission was detected in the photomultiplier at 520–550nm. APG-2 fluorescence emission was detected in the photomultiplier at 530–550nm. Images were analysed with Image J software (NIH, USA) to calculate cell fluorescence based on integrated density. The background signal was measured from an empty region with a similar size and subtracted from the whole-cell signal to obtain relative total cell fluorescence values (Bonales-Alatorre *et al.*, 2013b). Relative total cell K^+^, Ca^2+^, and Na^+^ concentration data shown in Figs 3, 5, and 7 were divided by 1000.

The cytosolic Ca^2+^ concentration in Arabidopsis root cells was also determined using the fluorescent dye Calcium Green. For image analysis, several lines were drawn across the region of interest as shown in Supplementary Fig. S2A in an appropriate zone with Leica Application Suite X software (Leica Microsystems). Continuous fluorescence was quantified in arbitrary units by LAS AF software based on intensity and plotted in an Excel file (Supplementary Fig. S2B) (Wu *et al.*, 2015).

### Statistical analysis

Statistical analysis was performed using IBM SPSS Statistics 21 (IBM, New York, USA). All data in the figures are given as means ±SE. The significant differences in relative gene expression level, relative total cell K^+^, Ca^2+^, and Na^+^ concentration, and phenotype were compared using Duncan’s multiple range test. Different lower case letters represent a significant difference between genotypes and treatments at *P<*0.05. The comparison of cytosolic Ca^2+^ concentration in different genotypes was done by paired samples *t*-test. The significance levels are *P<0.05, **P<0.01, and ***P<0.001.

## Results

### Hypoxia-induced changes in Ca^2+^ transporter transcript levels in Arabidopsis roots

**Fig. 1.**
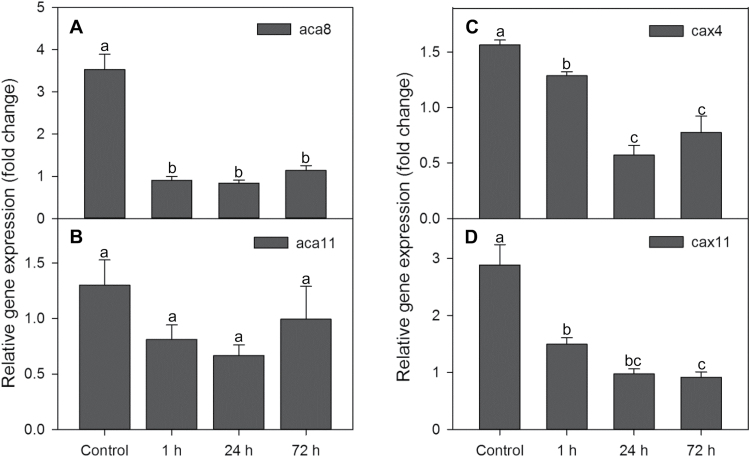
Relative expression of *ACA8*, *ACA11*, *CAX4*, and *CAX11* in roots of the Arabidopsis wild type (WT) under normoxic or hypoxic treatments for 1, 24, and 72h. *RPB2* was used as a reference gene. Data are the mean ±SE (*n*=3 separate experiments each involving 3–5 biological replicates). Different lower case letters indicate significant differences between treatments at *P*<0.05.

We investigated the effects of hypoxia on transcript levels of *ACA8*, *ACA11*, *CAX4*, and *CAX11* in the roots of Arabidopsis WT after 1, 24, and 72h of hypoxia treatment (Fig. 1). With the exception of *ACA11*, all genes were down-regulated after 1h of hypoxia treatment (Fig. 1). Hypoxia had the highest impact on transcript levels of *ACA8*, *CAX4*, and *CAX11* in Arabidopsis roots at 24h, with some transcripts reduced to only 33% of the control. Extending the hypoxia period to 72h produced no significant differences in expression levels of these genes in roots as compared with the 24h treatment.

### Hypoxia-induced K^+^ distributions show tissue- and zone-specific responses in roots of Arabidopsis mutants

**Fig. 2.**
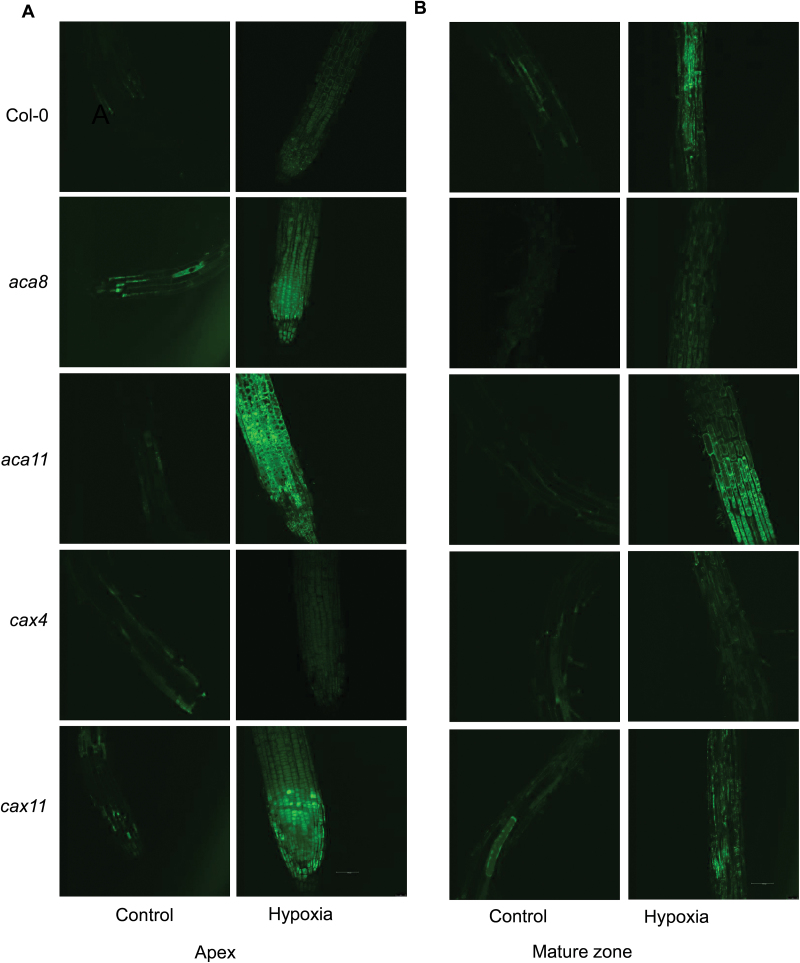
Distribution of K^+^ in root meristem, elongation, and mature zone in Arabidopsis wild type (Col-0), *aca8*, *aca11*, *cax4*, and *cax11* under normoxic or hypoxic treatments for 24h. (A) Representative images of the root apex in control and hypoxic treatment. (B) Representative images of root mature zone in control and hypoxic treatment. The relative K^+^ concentration in roots of 10-day-old seedlings was visualized using a confocal imaging system with Asante Potassium Green-2 fluorescent dye. One out of nine typical images is shown for each line. Scale bar=50 µm.

We studied changes of cellular K^+^ concentration in functionally different root zones (meristem, elongation, and mature) and tissues (epidermis and stele) in roots of Arabidopsis WT and four Ca^2+^ transporter mutants after 24h of hypoxia treatment (Figs 2, 3). In root epidermis, hypoxia led to an increase in the cellular K^+^ concentration in the root meristem but a decrease in the mature zone cells in WT Col-0 plants (Fig. 3A). In the root stele, the cellular K^+^ concentration was increased by 1.5- to 2-fold in both apex (elongation) and mature root tissues (Fig. 3B). Mutant plants showed similar trends to the WT, but with several exceptions. Hypoxia caused a dramatic increase in K^+^ concentration in meristem and stelar tissue in the elongation zone of roots of *aca8*, *aca11*, and *cax11* (Fig. 3), but led to a significant decrease in K^+^ concentration in these cell types in *cax4* (Fig. 3B). Interestingly, elongation zone cells in the epidermis in *cax4* had a significant decrease in K^+^ concentration after hypoxia, while there was no significant difference among the other four genotypes. Compared with the control, there was a 20% decrease in K^+^ in the mature zone in root epidermis in the WT and a 50% decrease in K^+^ in *aca8*, and no significant difference among the other lines (Fig. 3A; Table 1). Also in the stelar cells of mature zones, after 24h of hypoxia treatment, the K^+^ concentration increased dramatically in theWT, *aca11*, *cax4*, and *cax11*, but was not changed in *aca8* (Fig. 3B).

**Table 1.** Effects of 24h of hypoxia on quantitative characteristics of K^+^, Ca^2+^, and Na^+^ concentration measured from the meristem, elongation epidermal cell, elongation stelar cell, mature epidermal cell, and mature stelar cell in Arabidopsis wild type (WT), and mutants aca8, aca11, cax4, and cax11 (ratio of hypoxia ion concentration to control ion concentration)

		Meristem	Elongation epidermis	Mature epidermis	Elongation stele	Mature stele
WT	K^+^	1.5	1.0	0.8	1.7	1.5
	Ca^2+^	0.6	1.4	3.3	1.0	2.7
	Na^+^	1.1	0.9	1.1	1.5	3.7
*aca8*	K^+^	2.1	1.2	0.5	2.0	1.3
	Ca^2+^	0.8	1.0	1.0	1.1	4.1
	Na^+^	1.3	1.0	1.1	1.8	4.5
*aca11*	K^+^	2.8	1.2	1.2	2.3	2.2
	Ca^2+^	0.8	0.7	1.3	1.0	10.8
	Na^+^	2.6	2.2	2.2	2.9	16.1
*cax4*	K^+^	0.8	0.6	0.9	0.7	1.4
	Ca^2+^	1.0	0.6	1.7	0.8	3.3
	Na^+^	1.4	2.0	1.1	2.7	3.3
*cax11*	K^+^	2.0	0.9	1.1	1.5	1.4
	Ca^2+^	1.0	0.6	1.3	1.2	5.6
	Na^+^	2.4	1.5	1.0	1.6	3.4

Data are the ratio of hypoxia over control. Measurements were collected from the meristem, elongation zone epidermal cell, elongation zone stele cell, mature epidermal cell, and mature stele cell of five Arabidopsis lines.

### Hypoxia induces an elevation of Ca^2+^ in the mature root zone

**Fig. 4.**
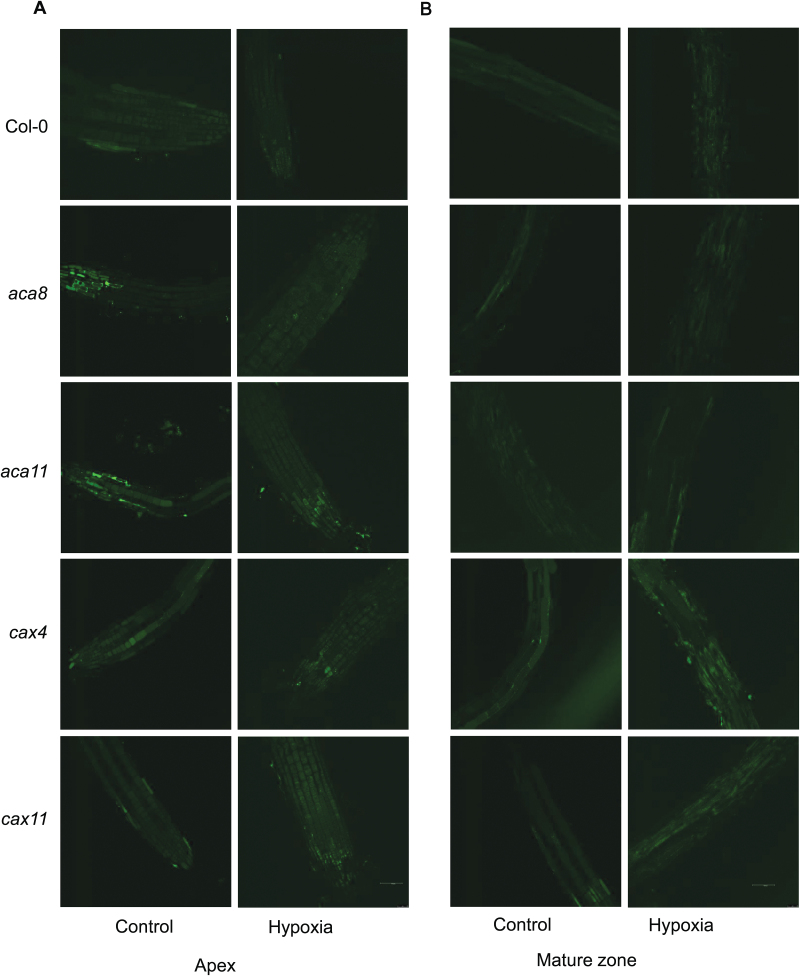
Distribution of Ca^2+^ in the root meristem, elongation, and mature zone in Arabidopsis wild-type (Col-0), *aca8*, *aca11*, *cax4*, and *cax11* under normoxic or hypoxic treatments for 24h. (A) Representative images of the root apex in control and hypoxic treatment. (B) Representative images of the root mature zone in control and hypoxic treatment. The relative Ca^2+^ concentration in roots of 10-day-old seedlings was visualized using a confocal imaging system with Calcium Green fluorescent dye. One out of nine typical images is shown for each line. Scale bar=50 µm.

**Fig. 5.**
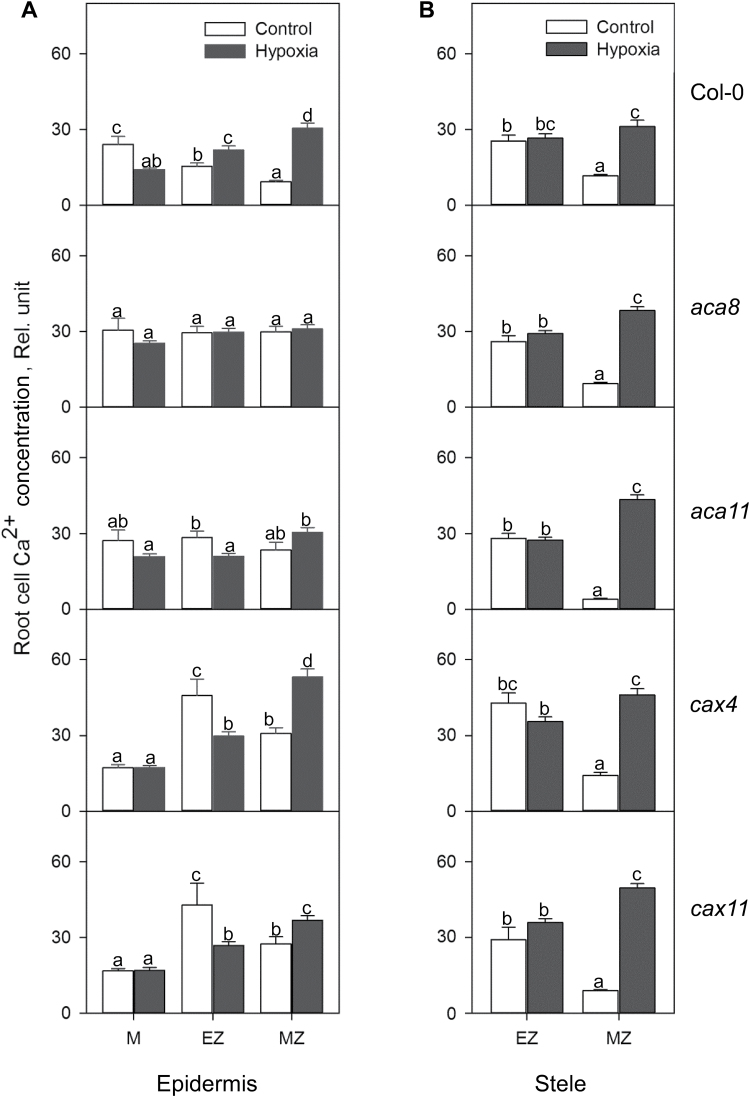
Relative Ca^2+^ concentration in the meristem, elongation, and mature zone in root epidermis (A) and stele (B) of Col-0, *aca8*, *aca11*, *cax4*, and *cax11* under normoxic or hypoxic treatments for 24h. The relative Ca^2+^ concentration was calculated by the fluorescence integrated density using Image J software. Data are the mean ±SE (*n*=30–40 cells from one individual plant with at least nine replicate plants). Different lower case letters indicate significant differences at *P*<0.05.

A clear difference in Ca^2+^ concentration was found in cells of the meristem, elongation zone, and mature zone after 24h of hypoxia treatment, showing genotypic difference after O_2_ deprivation (Figs 4, 5). In the root meristem (Fig. 5A), epidermal cells of WT plants showed a 40% loss of Ca^2+^ concentration under 24h of hypoxic conditions. However, no significant changes in Ca^2+^ concentration were detected in *aca8*, *aca11*, *cax4*, and *cax11* mutants compared with normoxic conditions (Fig. 5A; Table 1). In epidermal cells of the elongation zone (Fig. 5A), the Ca^2+^ concentration in the WT was much higher under hypoxic conditions, while a 30–40% decrease was measured in *aca11*, *cax4*, and *cax11* mutants, and no changes in *aca8*. Epidermal cells in the mature zone showed a dramatic increase in Ca^2+^ level in the WT (3.3-fold) and *cax* mutants (a 1.3- to 1.7-fold increase) and no changes in the *aca* mutants (Fig. 5A; Table 1). Comparing the stelar cells in the elongation and mature zones, hypoxia had no significant effect on Ca^2+^ in the elongation zone of any genotype, but resulted in marked increases in Ca^2+^ for stelar cells in the mature zone of *aca11*, with a 10.8-fold rise of Ca^2+^ compared with the control and a 5.6-fold increase in *cax11*, while the increase was only 2.7-fold in the WT (Fig. 5B).

### Combined hypoxia and salinity stress cause higher Na^+^ accumulation in stelar cells of the elongation zone and mature zone

**Fig. 6.**
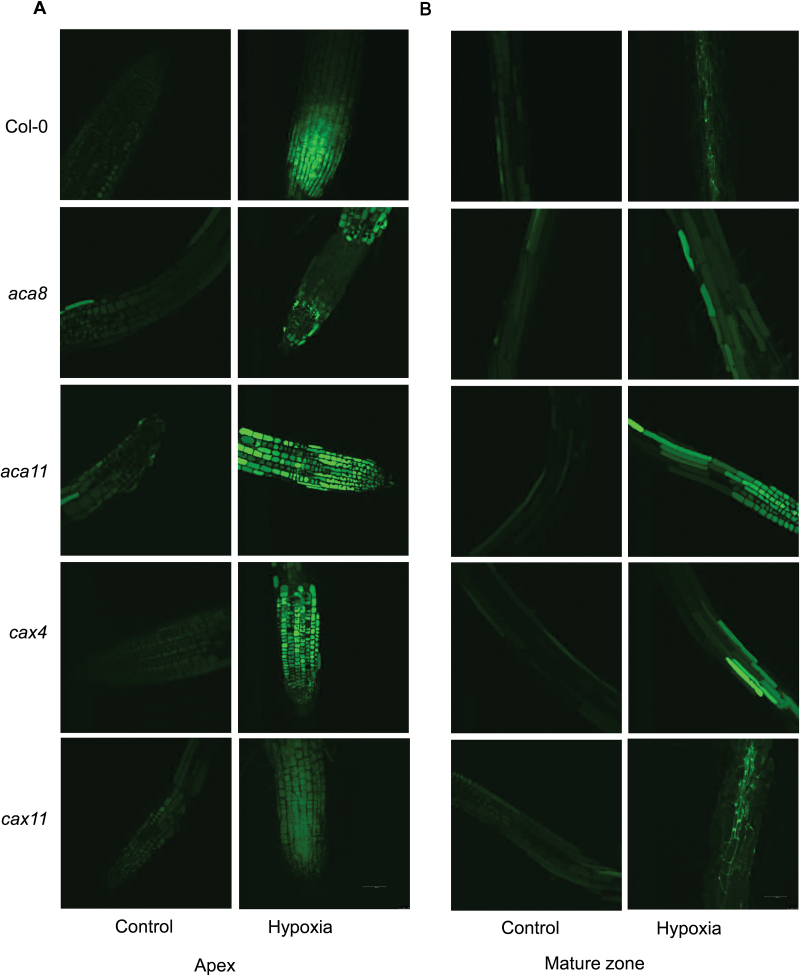
Distribution of Na^+^ in the root meristem, elongation, and mature zone in Arabidopsis wild type (Col-0), *aca8*, *aca11*, *cax4*, and *cax11* under normoxic or hypoxic treatments for 24h. (A) Representative images of the root apex in control and hypoxic treatment. (B) Representative images of the root mature zone in control and hypoxic treatment. The relative Na^+^ concentration in roots of 10-day-old seedlings was visualized using a confocal imaging system with CoroNa Green fluorescent dye. One out of nine typical images is shown for each line. Scale bar=50 µm.

**Fig. 7.**
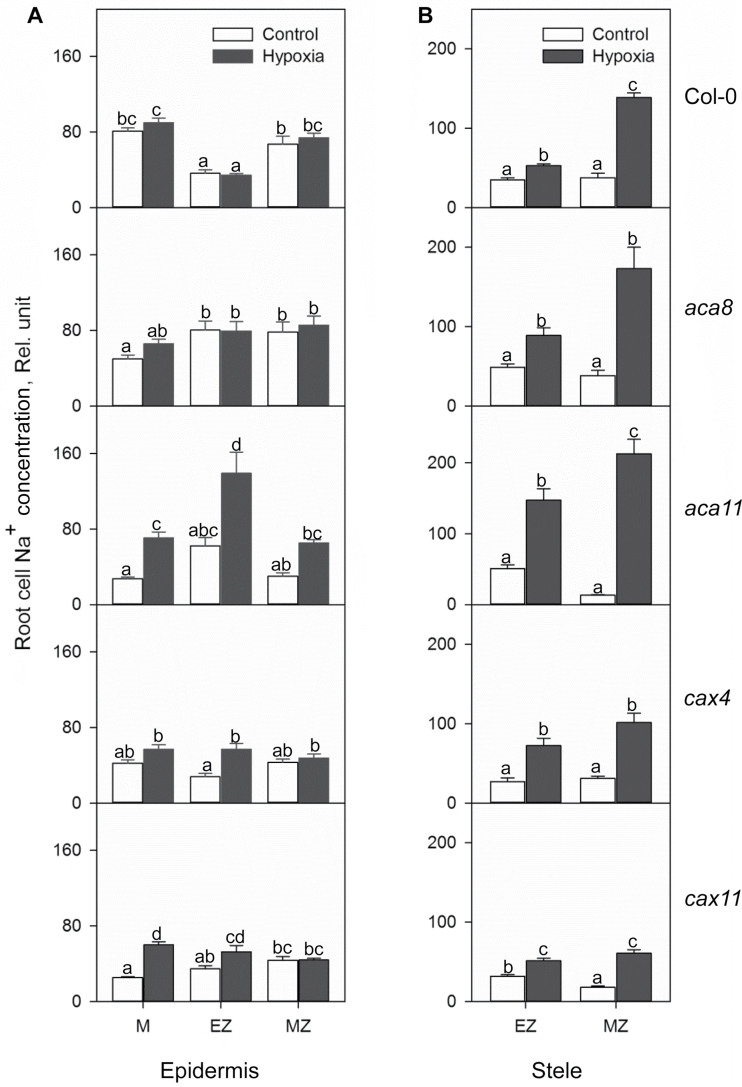
Relative Na^+^ concentration in the meristem, elongation, and mature zone in root epidermis (A) and stele (B) of Col-0, *aca8*, *aca11*, *cax4*, and *cax11* under normoxic non-saline or combined salinity and hypoxia treatments for 24h. The relative Na^+^ concentration was calculated by the fluorescence integrated density using Image J software. Data are the mean ± SE (*n*=30–40 cells from one individual plant with at least nine replicate plants). Different lower case letters indicate the significant difference at *P*<0.05.

NaCl treatment (50mM) with hypoxia caused dramatic increases in the Na^+^ concentration, especially in the root stele in both the elongation zone and mature zone (Fig. 7B). *aca11* showed a huge 16.1-fold increase in Na^+^ concentration in the mature zone in the root stele, while the changes for the WT and other mutants were from 3.3- to 4.5-fold higher than in the control. Also in stele, the Na^+^ concentration increased moderately from 1.5- to 2.9-fold in the elongation zone of all these genotypes (Fig. 7B). Hypoxia and salinity stress caused complex changes in the root epidermis (Figs 6, 7A). In root meristem cells, no significant differences of Na^+^ accumulation were found among WT, *aca8*, and *cax4*, while there were 2.6- and 2.4-fold higher levels of Na^+^ in *aca11* and *cax11*, respectively (Fig. 7A; Table 1). In epidermal cells of the elongation zone, there were no changes of Na^+^ distribution in the WT and *aca8*, but 2.2-, 2-, and 1.5-fold increases were found in *aca11*, *cax4*, and *cax11*. Interestingly, in the mature zone in the root epidermis, all the five genotypes showed no significant Na^+^ concentration changes compared with the control (Fig. 7A).

### More Ca^2+^ is accumulated in the cytosol of stelar cells in the mature zone

**Fig. 8.**
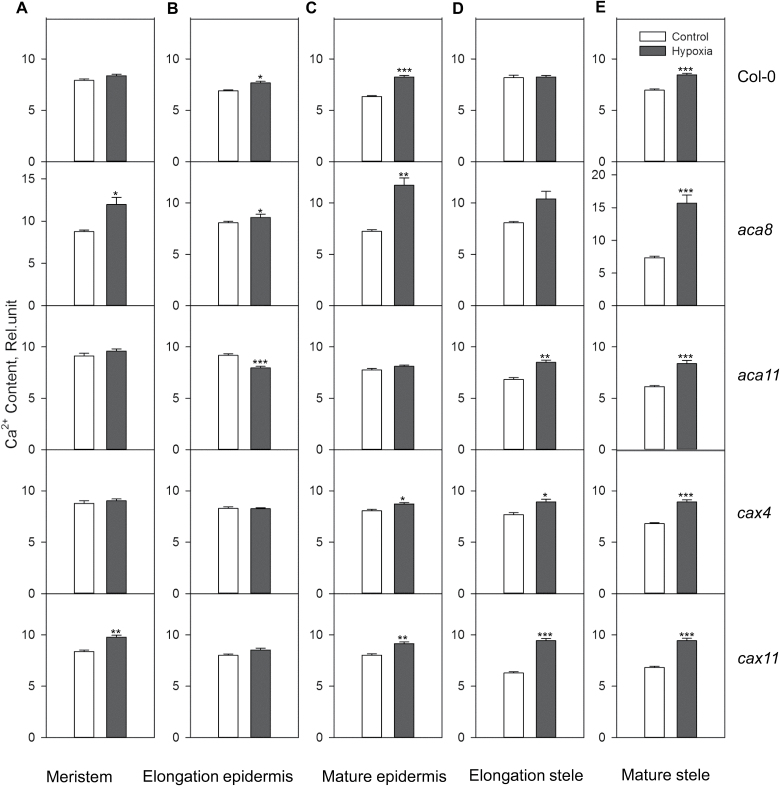
Relative Ca^2+^ concentration in the cytosol in the meristem, elongation, and mature zone in root epidermis and stele of Col-0, *aca8*, *aca11*, *cax4*, and *cax11* under normoxic or hypoxic treatments for 24h. Data are the mean ±SE (*n*=60 cells from one individual plant with at least nine replicate plants). Asterisks indicate significant differences at *P<0.05, **P<0.01, and ***P<0.001.

Hypoxic stress-induced changes in the [Ca^2+^]_cyt_ have been rarely considered in the context of the tissue-specific expression and regulation of appropriate Ca^2+^ transporters. Significantly (*P<*0.01) higher quantities of Ca^2+^ were accumulated in the cytosol of meristematic cells in *aca8* and *cax11* after 24h of hypoxia, while no changes were observed in the WT, *aca11*, and *cax4* (Fig. 8A). In the elongation zone in root epidermis, [Ca^2+^]_cyt_ increased significantly in the WT (*P<*0.05) and *aca8* (*P<*0.05) and highly significantly in *aca11* (*P<*0.001), but no changes were found in *cax4* and *acax11* (Fig. 8B). Epidermal cells in the mature zone showed significant elevation in [Ca^2+^]_cyt_ in all genotypes except *aca11* (Fig. 8C). Stelar root cells in the elongation zone had a higher Ca^2+^ concentration in the cytosol under hypoxic conditions in three of the mutants but not in the WT and *aca8* (Fig. 8D). In stelar cells of the mature zone, a significant (*P<*0.001) increase in the [Ca^2+^]_cyt_ was found in all the five genotypes (Fig. 8E).

### A loss of *CAX11* results in a sensitive phenotype to waterlogging stress

**Fig. 9.**
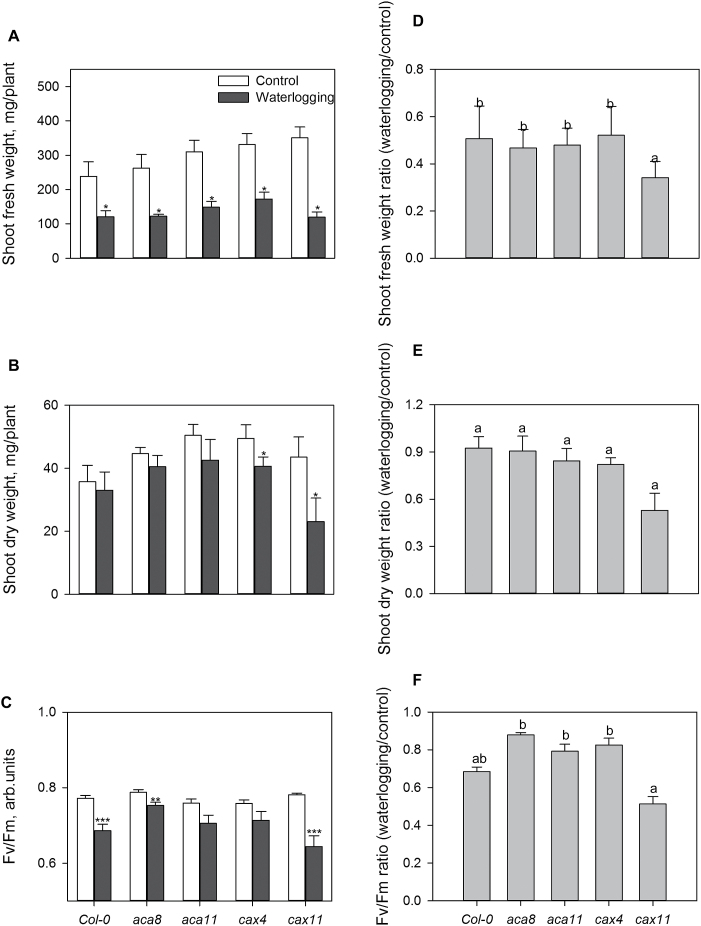
Effects of waterlogging stress on growth and maximum photochemical efficiency of PSII (*F*_v_/*F*_m_) of 6-week-old Arabidopsis plants. Three-week-old Arabidopsis plants were subjected to waterlogging treatment with water maintained at 0.5cm above the soil surface for 3 weeks. *F*_v_/*F*_m_ (D) was measured after 3 weeks of treatment and plants were used for morphological (A) and biomass analysis (B, C). Data are the mean ±SE (*n*=6). The ratio of shoot fresh weight loss (E), shoot dry weight loss (F), and *F*_v_/*F*_m_ loss (G) were calculated.

Growth of the Arabidopsis WT and all the mutants was significantly (*P*<0.05) reduced after 3 weeks of waterlogging stress (Fig. 9). The observed decline in the shoot fresh weight, dry weight, and shoot *F*_v_/*F*_m_ was highly genotype specific, with *cax11* more sensitive to waterlogging stress than the other genotypes. The knock-out mutant *cax11* showed the strongest growth phenotype and exhibited a 60% reduction in shoot fresh weight and 67% loss in shoot dry weight after 3 weeks of waterlogging. Meanwhile, the average waterlogging-induced reductions in the shoot fresh and dry weight in the WT and other mutants were only 42% and 18%, respectively (Fig. 9A, B, D, E). These results were consistent with the significant decrease in the maximal photochemical efficiency of PSII (*F*_v_/*F*_m_ chlorophyll fluorescence values) in leaves of *cax11* (Fig. 9C, F).

## Discussion

### Ca^2+^ efflux systems play important roles in hypoxic stress

A higher concentration of cytosolic Ca^2+^ can cause cytotoxicity owing to the precipitation of Ca^2+^ with phosphate and other anions, or binding to negatively charged macromolecules in the cytosol (Williams, 2006); it may also interfere with the cell’s ability to respond to various stimuli (Bose *et al.*, 2011). To restore the low resting concentration in the cytosol, Ca^2+^ has to be transported out against an electrochemical gradient into the apoplast, or into intracellular organelles by Ca^2+^-ATPases and/or Ca^2+^/H^+^ antiporters. It is suggested that Ca^2+^-ATPases with high-affinity (*K*_m_=0.1–2 µM) and low-capacity transporters are responsible for termination of [Ca^2+^]_cyt_ signalling; while Ca^2+^ exchangers with low-affinity (*K*_m_=10–15 µM) and high-capacity transporters may be involved in the removal of [Ca^2+^]_cyt_ when its elevations are high (Sze *et al.*, 2000; Bose *et al.*, 2011; Shabala *et al.*, 2015). Recently, CAX11 was reclassified as CCX5 (Cation Calcium eXchanger) due to higher homology to mammalian K^+^-dependent Na^+^/Ca^2+^ antiporters which catalyse the electrogenic countertransport of four Na^+^ for one Ca^2+^ and one K^+^ (Dong *et al.*, 2001; Cai and Lytton, 2004; Shigaki and Hirschi, 2006). Expression of AtCCX5 in a yeast mutant restored its growth in low K^+^ (0.5mM) medium and also suppressed its Na^+^ sensitivity (Zhang *et al.*, 2011). These findings suggest that CAX11 not only has the ability to remove Ca^2+^ into the vacuole but also plays a role in Na^+^ transportation. In the present study, *cax11* was more sensitive to 3 weeks of waterlogging stress than the WT and other mutants tested (Fig. 9). We speculate that mutation of ACA8, ACA11, CAX4, and CAX11 in Arabidopsis potentially reduces the ability to terminate [Ca^2+^]_cyt_ signalling or to remove [Ca^2+^]_cyt_ disturbance of Ca^2+^ homeostasis. Knock out of the multiple functional CAX11 might lead to sensitivity to waterlogging stress.

Ca^2+^ efflux systems play important roles in stress tolerance (Qudeimat *et al.*, 2008; Bose *et al.*, 2011). The role of ACAs in shaping [Ca^2+^]_cyt_ signatures under salinity stress was first demonstrated in yeast (Anil *et al.*, 2008). Knocking out *AtACA4* and *AtACA2* resulted in an increased NaCl sensitivity, while their overexpression in Arabidopsis seedlings led to increased stress tolerance in comparison with WT plants (Geisler *et al.*, 2000). After 2–4h of low O_2_ treatment, *ZmCAP1* encoding Ca^2+^-ATPase in maize roots showed a 2- to 3-fold increase in expression levels (Subbaiah and Sachs, 2000). In contrast, 1h and 24h hypoxic treatments reduced the expression of *ACA8*, *CAX4*, and *CAX11* in WT roots to 33–50% of the control (Fig. 1), but not for *ACA11*. Reports on the functional role of ACA11 in plants are rather scarce, to say nothing of its modulation by hypoxic stress. ACA11 was identified as localized on the tonoplast and preferentially expressed in Ca-rich mesophyll; the simultaneous knock out of *ACA4* and *ACA11* does not appear to reveal a phenotype associated with perturbed apoplastic Ca^2+^ homeostasis compared with *cax1*/*cax3* (Conn *et al.*, 2011). In the current study, after 3 weeks of waterlogging stress, the *aca11* mutant did not show significant changes in shoot dry weight and maximum photochemical efficiency of PSII (chlorophyll fluorescence) compared with the control, consistent with the insignificant changes of *ACA11* expression in the WT, which suggests that ACA11 is unlikely to play an important role in hypoxic stress responses. In rice, Ca^2+^ was reported to act as a physiological transducer for the expression of the *alternative oxidase 1a* (*AOX1a*) gene under O_2_ deficiency (Tsuji *et al.*, 2000). Here we suggest that hypoxia induces a rapid elevation of Ca^2+^ concentration as a signal, which probably inhibits the expression of Ca^2+^ efflux transporters. The *Atcax1* mutant showed enhanced tolerance to freezing following cold acclimation (Catala *et al.*, 2003). In contrast, *Atcax3* has increased sensitivity to salt stress (Zhao *et al.*, 2008). In our present work, *cax11* was more sensitive to waterlogging than the WT (Fig. 9). Ca^2+^/H^+^ exchangers may be involved in resetting the [Ca^2+^]_cyt_ elevation following different kinds of stress induction with specific characteristics (Dodd *et al.*, 2010). The differential stress sensitivities of the *cax* mutants may be a consequence of specific responses by AtCAX1, AtCAX3, and AtCAX11 to the individual stresses (McAinsh and Pittman, 2009).

### Hypoxia stress leads to cell- and tissue-specific changes in K^+^, Ca^2+^, and Na^+^ homeostasis

Over the last few decades, increasing numbers of reports showed that plant stress tolerance is conferred by cell type-dependent processes (Roberts and Tester, 1995; Nylander *et al.*, 2001; Ahmad and Maathuis, 2014). In this study, hypoxia had significant impacts on tissue distributions and cellular concentrations of K^+^ and Ca^2+^ in roots of Arabidopsis WT and Ca^2+^ transporter mutants. The combined hypoxia and salinity stress also had significant effects on the distribution of Na^+^ in different tissues and cell types in Arabidopsis WT and Ca^2+^ transporter mutants. Correspondingly, O_2_ gradients have been found along both longitudinal (Pang *et al.*, 2006) and radial (Armstrong, 1979; Armstrong *et al.*, 1994; Gibbs *et al.*, 1998) root profiles under hypoxic stress. It was demonstrated that with the shoot in the air, different barley root zones had different O_2_ requirements for uptake from the medium, with higher O_2_ influx in the elongation zone than in the mature zone (Pang *et al.*, 2006). An excised maize root showed a steep O_2_ concentration decline from the outer layers of cells to the stelar cells when in hypoxic solution (Gibbs *et al.*, 1998), showing more severe hypoxic stress within the stele than in the cortex. Intact barley roots in deoxygenated stagnant solution and relying on internal O_2_ movement into and along the root aerenchyma had slightly higher O_2_ in various tissues in the subapical region than just behind the root tip. At the subapical region clear radial gradients in O_2_ were seen, with the stele having the lowest O_2_ concentration (Kotula *et al.*, 2015). Therefore, it is suggested that different cell types in roots with diverse access to, and potential sensitivity to, O_2_ operate differently under hypoxic stress. Our present data further support this suggestion that various cell types in different zones regulate concentrations of K^+^, Ca^2+^, and Na^+^ in distinct ways when faced with hypoxia.

Calcium is a universal secondary messenger response to abiotic and biotic stress (Chen *et al.*, 2010; Shabala *et al.*, 2011; Scherzer *et al.*, 2015), but the changes in Ca^2+^ in different tissue-specific cells in roots under hypoxia required elucidation. The interesting finding in our data is that in the WT and all of the four Ca^2+^ transporter mutants (*aca8*, *aca11*, *cax4*, and *cax11*), the stelar cells in the elongation zone showed no changes in Ca^2+^ concentration, while in the mature zone they had higher Ca^2+^ than in normoxic controls (Fig. 5). This situation is similar to the stelar cells in the mature zone which also had significant increases of [Ca^2+^]_cyt_ in these five genotypes when treated with hypoxia (Fig. 8E). There have been very few studies on the Ca^2+^ concentration changes in different tissue-specific cells in roots under hypoxia. Studies on Arabidopsis leaf showed that there is more Ca^2+^ accumulated in the vacuoles of mesophyll cells then in those of the epidermis (Conn *et al.*, 2011), which has been consistently observed across all eudicots so far examined. Conversely, the cereal monocots barley and wheat preferentially accumulate Ca^2+^ in epidermal cells and not within mesophyll or vascular bundle cells (Conn and Gilliham, 2010). In general, the simple presence or absence of ion transporters could not explain cell type-specific differences in ion accumulation. However, we might suggest that the higher concentration of Ca^2+^ observed in stelar cells in the mature zone than in stelar cells in the elongation zone in hypoxic roots provides evidence for the necessity for energetically expensive Ca^2+^ accumulation in the root stele tissue (Subbaiah and Sachs, 2003).

There are numerous studies reporting perturbation in intracellular K^+^ homeostasis in response to hypoxia in plants (Wiengweera and Greenway, 2004; Mugnai *et al.*, 2011; Barrett-Lennard and Shabala, 2013). Hypoxia increased K^+^ efflux in 6-day-old wheat roots due to membrane depolarization and the increase of membrane permeability of K^+^ (Pang and Shabala, 2010). In *Vitis riparia* roots, an anoxia-tolerant variety, severe K^+^ imbalance is avoided during anoxia stress by decreasing membrane permeability of K^+^; whereas *Vitis rupestris*, an anoxia-sensitive variety, had a strong decrease of K^+^ (Mancuso and Marras, 2006). Accordingly, in aerobic roots, xylem loading of ions from the external medium is achieved with energy provided by the plasma membrane H^+^-ATPase (De Boer and Volkov, 2003) through the depolarization-activated outwardly rectifying K^+^ (SKOR) channels. Since the stele is the first root tissue suffering O_2_ deficiency, the H^+^-ATPase activities in the xylem would be inhibited, leading to the closure of SKOR channels for a substantial decline of xylem K^+^ concentration (Colmer and Greenway, 2011). However, the present data in our experiment showed that there was more K^+^ accumulation in the stele than in the epidermis in both the elongation zone and mature zone in the WT, *aca11*, and *cax11*, which is contrary to the former theory. We speculate that the K^+^ accumulation in stele could probably flow via NORCs (non-selective outward-rectifying channels) (Chen *et al.*, 2007; Zepeda-Jazo *et al.*, 2008). Unfortunately, the molecular identity of NORCs (as other non-selective cation channels) remains elusive (Demidchik and Maathuis, 2007), and their expression levels cannot thus be thus directly measured. In addition, cells in hypoxic tissues would still have produced some ATP by oxidative phosphorylation when O_2_ was above zero, or from glycolysis linked to ethanolic fermentation (Kotula *et al.*, 2015).

Although waterlogging is a widespread stress in its own right, its impact on a plant performance is often confounded by soil salinity (Barrett-Lennard and Shabala, 2013). Even a brief period of root O_2_ deprivation might result in a prolonged Na^+^ accumulation even after return to normoxic conditions (Marschner, 2011). Thus, it was of interest to measure the Na^+^ concentration under both hypoxia and salinity conditions. After 24h of combined salt and hypoxic treatment, there was a significant increase in Na^+^ in the elongation zone stele and a dramatic increase in mature stelar cells in the WT and all of the four Ca^2+^ transporter mutants (Fig. 7B). H^+^-ATPase pumps are responsible for the maintenance of the highly negative membrane potential at the root plasma membrane, and the low energy in O_2_-deficient tissues leads to a substantial depolarization of the plasma membrane potential and the closure of SKOR, which is highly selective for K^+^ over Na^+^ (Dietz *et al.*, 2001; Koizumi *et al.*, 2011; Zeng *et al.*, 2014). In contrast, the NORC, transporting K^+^, Na^+^, and anions could open during hypoxia, and NORC is also considered as the main transporter for loading Na^+^ into the xylem (Wegner and Raschke, 1994; Wegner and DeBoer, 1997; Pang and Shabala, 2010), which probably explains the increase of Na^+^ in cells of both the elongation zone and mature stele. Interestingly, we also found that there was a 2.6-fold increase in Na^+^ concentration in mature stelar cells relative to elongation zone stelar cells under hypoxia, while there was no difference between the two kinds of cells in the WT under normoxic conditions (Supplementary Table S2). This may be suggestive that even under hypoxia there is still Na^+^ flow in the xylem from the root to the shoot via other pathways. Some studies found that protein kinase SOS2 (Salt Overly Sensitive 2)/CIPK24, involved in salt tolerance (Cheng *et al.*, 2004), could bind to the autoinhibitory domain of CAX1 to activate the Ca^2+^ transport into the yeast vacuole, making the cells tolerant to the high Ca^2+^ in the media. This result implies the co-regulation of CAX1 and the SOS1 antiporter by SOS2/CIPK24 in the signalling pathway for salinity tolerance (Shigaki and Hirschi, 2006). Therefore, a possible convergence of salinity and hypoxia tolerance via the link to Ca^2+^ signalling and Ca^2+^ efflux systems is highly likely. This question requires a detailed study in the future.

### Conclusions

The results presented here allow a new hypothesis concerning the hypoxic signal transduction in Arabidopsis roots linked with CAX and ACA Ca^2+^ transporter systems. The tendency in past research on hypoxic stress has been to focus on whole roots, but the key challenge now is to try to explain root function in terms of the different root zones (meristem, elongation, and mature), tissues (epidermis and stele), and specific cell types. In this study, unique cell types in different zones regulate concentrations of K^+^, Ca^2+^, and Na^+^ in distinct ways when faced with hypoxia. In the WT and all of the four Ca^2+^ transporter mutants, the stelar cells in the elongation zone showed no changes in Ca^2+^ concentration, while in the mature zone they had higher Ca^2+^ than normoxic controls. We also speculate that more K^+^ and Na^+^ accumulation in the stele than in the epidermis in both the elongation zone and mature zone could probably flow via NORC which could open during hypoxia. In addition, Ca^2+^ efflux systems and in particular CAX11 were shown to mediate plant Ca^2+^ homeostasis under hypoxic conditions, with an influence on waterlogging tolerance.

## Supplementary data

Supplementary data are available at *JXB* online.

Figure S1. Dose dependence of Calcium Green loading into five types of Arabidopsis root cells.

Figure S2. Illustration of the quantification procedure for Ca^2+^ distribution in the cytosol.

Table S1. The primers for quantitative RT-PCR experiments.

Table S2. Effect of 24h of hypoxia on Na^+^ relative concentrations in stelar cells of the elongation zone and mature zone in Arabidopsis WT, *aca8*, *aca11*, *cax4*, and *cax11* (ratio of Na^+^ concentration in mature stelar cells relative to elongation stelar cells under control or hypoxic conditions).

Supplementary Data
