# Supplementary material for: Tissue-specific root ion profiling reveals essential roles of the CAX and ACA calcium transport systems in response to hypoxia in Arabidopsis
Source: J Exp Bot. 2016 Feb 17;67(12):3747–62. doi: 10.1093/jxb/erw034 (PMC4896357; doi:10.1093/jxb/erw034)
Supplement: Supplementary Data [file supp_67_12_3747__index.html]

Tissue-specific root ion profiling reveals essential roles for the CAX and ACA calcium transport systems for hypoxia response in Arabidopsis — Tissue-specific root ion profiling reveals essential roles of the CAX and ACA calcium transport systems in response to hypoxia in Arabidopsis — Supplementary Data 

# Tissue-specific root ion profiling reveals essential roles of the CAX and ACA calcium transport systems in response to hypoxia in Arabidopsis

## Supplementary Data

Data files

- Supplementary\_tables\_S1\_S2\_figures\_S1\_S2.pdf - Supplementary Data
