# Supplementary material for: Tissue-specific root ion profiling reveals essential roles of the CAX and ACA calcium transport systems in response to hypoxia in Arabidopsis
Source: J Exp Bot. 2016 Feb 17;67(12):3747–62. doi: 10.1093/jxb/erw034 (PMC4896357; doi:10.1093/jxb/erw034)

Supplementary Data

Tissue-specific root ion profiling reveals essential roles of the CAX and ACA calcium transport systems for hypoxia response in *Arabidopsis*

Feifei Wang[1], Zhong-HuaChen[2], Xiaohui Liu[2,3], Timothy David Colmer[4], Meixue Zhou[1], Sergey Shabala[1,*]

[1]School of Land and Food, University of Tasmania, Hobart, Tasmania 7001,Australia

[2]School of Science and Health, Western SydneyUniversity, Penrith, NSW2751, Australia

[3]School of Chemical Engineering and Technology, Tianjin University, Tianjin 300072, China

[4]School of Plant Biology and Institute of Agriculture, The University of Western Australia, Crawley, WA, 6009, Australia

[*]Corresponding author; email:Sergey.Shabala@utas.edu.au

Running Title: calcium transportersin hypoxia responses

Supplementary Data

Supplementary Fig. 1. Dose-dependence of Calcium Green loading into five types of *Arabidopsis* root cells. *Arabidopsis* seedlings WT were incubated in Calcium Green dye solution for 3 h in the dark with dye concentration ranged from 0 to 50 µM. After loading, the fluorescent integrated density in specific root cells was measured by confocal scanning microscope and analysed by Image J software. Data are mean ± SE (n=30-40 cells from one individual plant with at least 9 replicate plants).

Supplementary Fig. 2. Illustration of the quantification procedure for Ca^{2+} distribution in cytosol. (A) Several lines are drawn across the interest region in an appropriate root zone. (B) Continuous fluorescent intensity in cytosol and vacuole was quantified in arbitrary units by LAS software and plotted in an Excel file. Scale bar=20µm.

Supplementary Table1. The primers for Quantitative RT-PCR experiments.

Supplementary Table2. Effect of 24 h of hypoxia on Na^+ relative concentrations in stelar cells of elongation zone and mature zone in *Arabidopsis* WT, *aca8*, *aca11*, *cax4* and *cax11*(ratio of Na^+ concentration in mature stelar cells relative to elongation stelar cells under control or hypoxic condition).

Table S1. List of primers for Quantitative RT-PCR

Name	Primer
ACA8-F	5'-TCGTAAACCAGATGAGAAGAACA-3'
ACA8-R	5'- ACCGATGCCAACACAGATAAG-3'
ACA11-F	5'-ATCTGCACGGATAAAACAGG-3'
ACA11-R	5'- GTTCGGATAGGTTCAACTGG- 3'
CAX4-F	5'- GTATTACAGGATGGGACTTCG-3'
CAX4 -R	5'-GTATTGGTTTCGGTTGAGGG-3'
CAX11-F	5'- CTTCGAATCCCACAAAAAGTTC-3'
CAX11-R	5'- AAAGTAAACCAGATGGGGGAGT-3'
RPB2-F	5'- TTCCCCGTTCCGATAACT-3'
RPB2-R	5'- ATGCTCTGCCGTCCACC-3'

Table S2. Effect of 24 h of hypoxia on Na^+ relative concentrations in stelar cells of elongation zone and mature zone in *Arabidopsis* WT, *aca8*, *aca11*, *cax4* and *cax11*.

MZ/EZ	Control	Hypoxia
WT	1.1	2.6
aca8	0.8	2.0
aca11	0.3	1.4
cax4	1.2	1.4
cax11	0.6	1.2

Note: Data are the ratio of Na^+ concentration in mature stelar cells relative to elongation stelar cells under control or hypoxic condition. Measurements were collected from elongation stele cell and mature stele cell of five *Arabidopsis* lines.

Supplementary Fig. 1

A

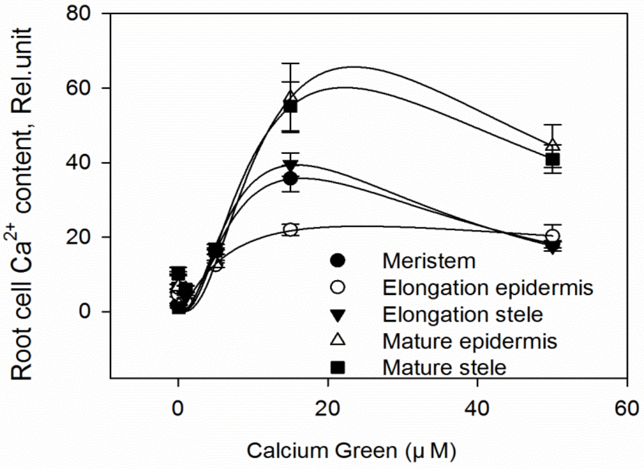

Supplementary Fig. 2

A

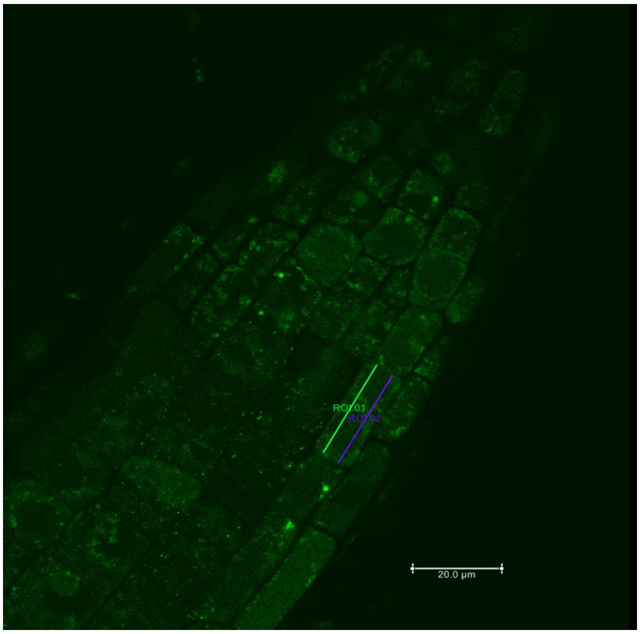

B

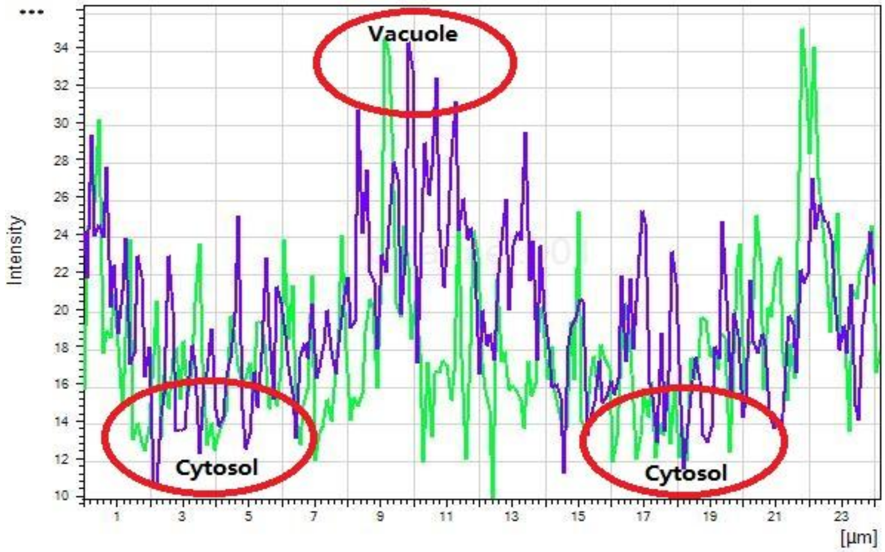

Supplement: Supplementary Data [file supp_erw034_Supplementary_tables_S1_S2_figures_S1_S2.pdf]
